# Short- and Long-Term Outcomes After MAIA-Guided Biofeedback Training in 346 Patients with Low Vision: A Retrospective Cohort Study

**DOI:** 10.3390/brainsci16101089

**Published:** 2026-10-10

**Authors:** Mariana A. M. Misawa, Aljeena Rahat Qureshi, Ghaliah Nsour, Tina Felfeli, Jack E. Teplitsky, Samuel N. Markowitz, Monica Daibert Nido

**Affiliations:** 1 Department of Ophthalmology and Vision Sciences, Toronto Western Hospital, University Health Network, 399 Bathurst St., Toronto, ON M5T 2S8, Canadaaljeena.qureshi@mail.utoronto.ca (A.R.Q.); tina.felfeli@mail.utoronto.ca (T.F.);; 2 Department of Medicine, Queen’s University, Etherington Hall, 3022-94 Stuart Street, Kingston, ON K7L 3N6, Canada; 3 Krembil Research Institute, 60 Leonard Avenue, Toronto, ON M5T 2S8, Canada; 4 Donald K. Johnson Eye Institute, Krembil Discovery Tower, 60 Leonard Avenue, Toronto, ON M5T 2S8, Canada

**Keywords:** biofeedback, neuroplasticity, vision, perceptual learning, microperimetry, low vision

## Abstract

**Highlights:**

**What are the main findings?**

- MAIA-guided biofeedback training was associated with significant improvements in fixation performance and short-term distance and near visual acuity in a large, heterogeneous cohort of 346 patients with low vision.
- In the overall cohort, improvements in distance and near visual acuity were not statistically significant at long-term follow-up. An exploratory analysis suggested that progression of the underlying ocular disease may contribute to attenuation of visual acuity gains over time.

**What are the implications of the main findings?**

- Microperimetric biofeedback may represent a clinically useful, non-invasive rehabilitation strategy across a broad range of ocular and neurological causes of visual impairment.
- These findings support prospective controlled studies to confirm treatment efficacy, identify patients most likely to benefit, and determine the durability and optimal protocols for biofeedback training.

**Abstract:**

Purpose: To describe short- and long-term visual outcomes after MAIA microperimetry biofeedback training (BT) in a low-vision rehabilitation cohort. Methods: Retrospective study of patients who completed BT between 2017 and 2023 (4.1 ± 0.6 sessions). The MAIA device recorded P1 and P2 parameters, defined as the percentage of fundus-tracked fixation points falling within device-defined circular windows centered on the trained retinal locus (TRL). Outcomes: baseline, short-term follow-up (<6 months), and long-term follow-up (≥6 months): P1, P2, best-corrected distance visual acuity (BCVA), near visual acuity, fixation stability (BCEA), mean retinal sensitivity (dB), and reading speed (wpm) MNRead app. Results: A total of 346 participants were included. Median P1 increased from 21.5% at Session 1 to 32.0% at Session 5, and P2 increased from 66.0% to 81.5%. Distance and near visual acuity improved significantly at short-term follow-up but were not significantly different from baseline in the overall cohort at long-term follow-up. Reading speed, BCEA, and mean retinal sensitivity did not show significant longitudinal changes. In an exploratory secondary analysis excluding eyes with worsening BCVA, distance and near visual acuity remained significantly improved at long-term follow-up. Conclusions: MAIA-guided BT was associated with improved within-training fixation performance and short-term visual acuity in this heterogeneous retrospective cohort. Long-term improvement was not demonstrated in the overall cohort; exploratory analyses suggest that underlying disease progression may influence long-term outcomes. Prospective controlled studies are required to determine durability and efficacy.

## 1. Introduction

Low vision substantially limits reading, medication management, face recognition, navigation, and safe mobility, yet many patients are not offered structured rehabilitation that teaches efficient use of their residual vision [1,2]. In individuals with central vision loss, fixation frequently shifts from the damaged fovea to an eccentric preferred retinal locus (PRL), which functions as a “pseudo-fovea.” However, the spontaneously adopted PRL is not always optimally positioned for visual tasks and is often associated with unstable fixation, reducing visual efficiency [3]. Clinically, two characteristics of the PRL are particularly important: its retinal location and its fixation stability, commonly quantified by the 63% bivariate contour ellipse area (BCEA). Although PRLs typically develop near the border of a scotoma, multiple PRLs or task-dependent fixation strategies may emerge in some patients.

Microperimetry-guided biofeedback training (BT) has become an important component of contemporary low vision rehabilitation (LVR). By combining real-time retinal tracking with auditory and visual feedback, BT trains patients to improve fixation stability and to optimize—or relocate—their fixation toward a preferred or trained retinal locus (PRL/TRL) located in an area of greater retinal sensitivity and more favorable retinal span [4]. The macular integrity assessment (MAIA) microperimeter provides an integrated platform for identifying PRLs, quantifying fixation stability, measuring retinal sensitivity, and delivering targeted training within routine clinical practice.

Evidence supporting microperimetry-guided BT has accumulated across several retinal and neuro-ophthalmic conditions. Small prospective and retrospective studies have demonstrated improvements in distance and near visual acuity, fixation stability, reading performance, and patient-reported visual function. Functional neuroimaging studies in Stargardt disease further suggest that these behavioral gains may be accompanied by cortical reorganization, with increased activation of visual cortical areas following training [5,6,7,8]. Together, these findings support the biological plausibility of BT; however, most published studies remain limited by relatively small sample sizes, single-disease populations, and short follow-up periods, leaving important questions regarding its effectiveness and durability in routine clinical practice.

The present study addresses these gaps by evaluating one of the largest real-world cohorts of patients undergoing MAIA-guided biofeedback training. We retrospectively analyzed 346 patients with a broad spectrum of low vision diagnoses to quantify short- and long-term changes in distance and near visual acuity, fixation stability (63% BCEA), retinal sensitivity, and reading speed following BT. By reflecting routine clinical workflows across a heterogeneous patient population, this study aims to provide clinically relevant evidence to guide patient selection, counseling, treatment scheduling, and program development in low vision rehabilitation.

## 2. Methods

### 2.1. Study Design

We conducted a retrospective cohort study of patients receiving microperimetry-guided BT in the Low Vision Rehabilitation services of the Department of Ophthalmology & Vision Sciences (DOVS), University of Toronto. Care was delivered in routine practice at two clinics (Toronto Western Hospital and CNIB Low Vision). Clinical assessment data and BT session logs were abstracted from the medical records. The study adhered to the Declaration of Helsinki and was approved by the University Health Network Research Ethics Board (number 23-5412) in Toronto, Canada, and all the patients signed an informed consent allowing their data to be included in the study.

### 2.2. Participants

All consecutive patients who initiated BT between 1 January 2017 and 22 June 2023 were screened. Inclusion criteria were age ≥5 years and availability of a baseline (pre-BT) assessment and ≥1 post-BT follow-up within a predefined window. Exclusion criteria were incomplete BT protocol (<3 sessions), missing primary outcome at all post-BT timepoints, or inadequate microperimetry quality (operator-flagged unreliable fixation or artifact precluding BCEA computation). Of the 406 patients screened, 22 were excluded because of an incomplete BT protocol, 19 because they were lost to follow-up, and 19 because records were inaccessible, leaving 346 patients for inclusion in the study. For patients with worsening BCVA at long-term follow-up, available clinical records, microperimetry, and retinal imaging were reviewed for evidence of progression of the underlying ocular disease. Disease progression was assessed, when data were available, using measures independent of BCVA, including reduction in the number of seeing points on microperimetry, decline in mean retinal sensitivity in decibels, and, in eyes with geographic atrophy, enlargement of the atrophic area on fundus infrared or color photography. These measures were used to provide anatomic and functional context for the observed visual acuity decline rather than to define worsening based on BCVA alone. These patients remained included in the primary analysis of the overall cohort. An exploratory secondary analysis was subsequently performed after excluding eyes with worsening BCVA to characterize visual outcomes among participants whose visual acuity remained stable or improved during follow-up. Because subgroup membership was defined partly by the outcome itself, this analysis was considered exploratory and was not used to infer treatment efficacy or durability.

### 2.3. Biofeedback Training Protocol

BT was delivered using the biofeedback module of the MAIA microperimeter (Centervue, Padova, Italy). The intervention used real-time fundus tracking with auditory and visual feedback to train fixation toward a selected trained retinal locus (TRL). The TRL was selected individually based on the patient’s microperimetry findings, retinal sensitivity, and fixation pattern, with the aim of identifying a retinal location with preserved sensitivity that could provide more stable and functionally useful fixation. TRL selection was therefore individualized according to retinal function and fixation characteristics rather than determined by a single standardized location-selection protocol. For patients with macular or visual-field loss, the therapeutic goal was relocation and consolidation of fixation at a retinal location with better sensitivity, a larger usable visual span, and greater fixation stability. For patients with nystagmus, the therapeutic goal was improvement of fixation stability rather than relocation.

Each BT session consisted of 20 min of active training. The total duration of a training visit could exceed 20 min when rest breaks were required; however, these pauses were not included in the active training time. Patients received three to five training sessions (mean 4.1 ± 0.6). The number of sessions varied because the clinical protocol evolved over the study period: the protocol initially consisted of three sessions, was subsequently increased to four sessions, and, more recently, was extended to five sessions in some cases. Thus, the number of sessions was determined primarily by the clinical protocol in place at the time of treatment rather than by a standardized patient-specific criterion. Rest breaks were provided as needed.

### 2.4. Outcomes

**Figure 1:**
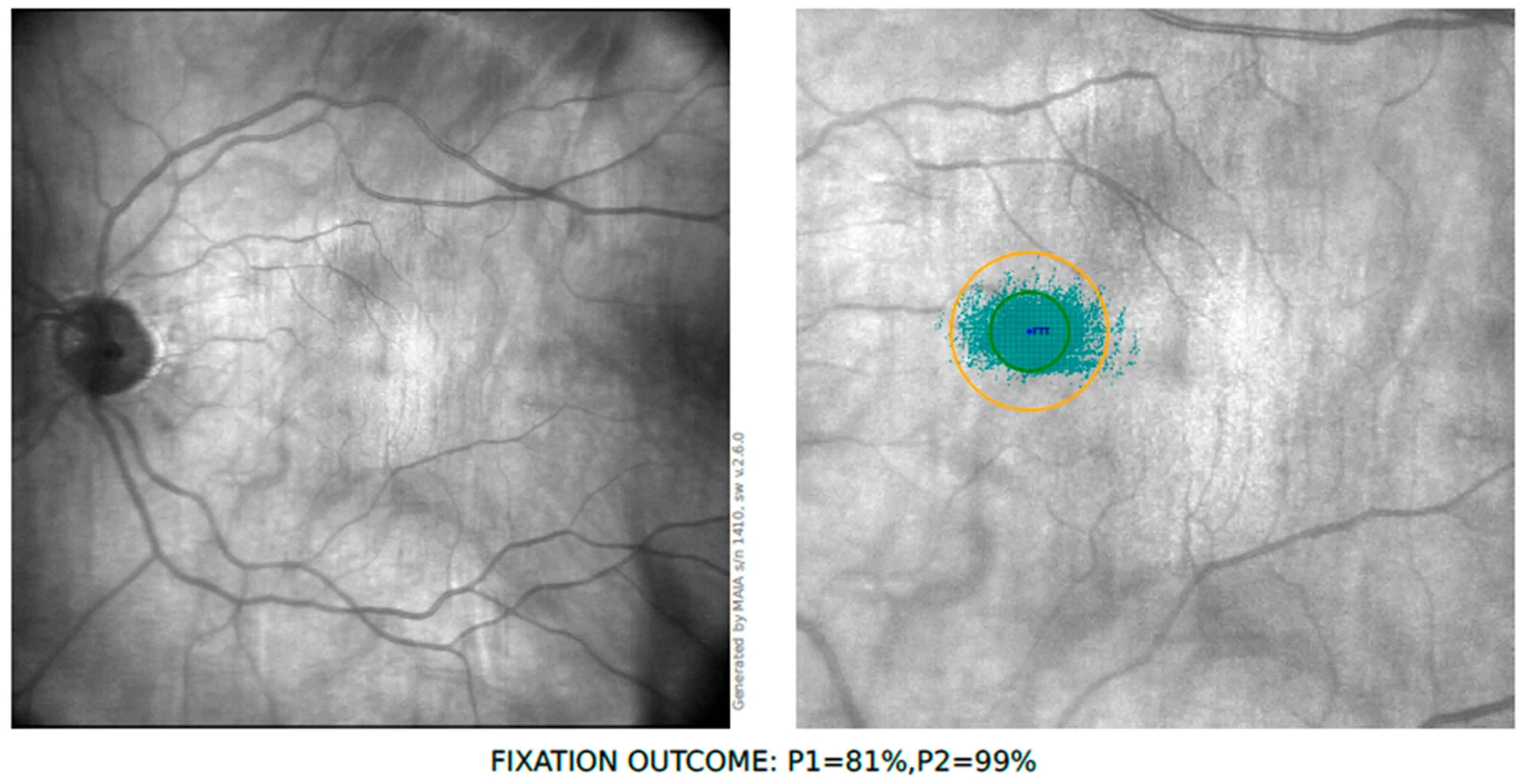
In-session fixation control during biofeedback training. The functional training target (FTT) corresponds to the selected trained retinal locus (TRL). Fixation performance was quantified using MAIA-derived metrics. P1 (green circle) represents the percentage of fundus-tracked fixation points within a 1° radius (2° diameter) centered on the TRL. P2 (yellow circle) represents the percentage of fixation points within the broader target window (2° radius; 4° diameter). In this example, fixation (represented by the green dots) was highly stable during training, with 81% of fixation points within P1 and 99% within P2, indicating consistent adherence to the intended fixation area throughout the session.

**Figure 2:**
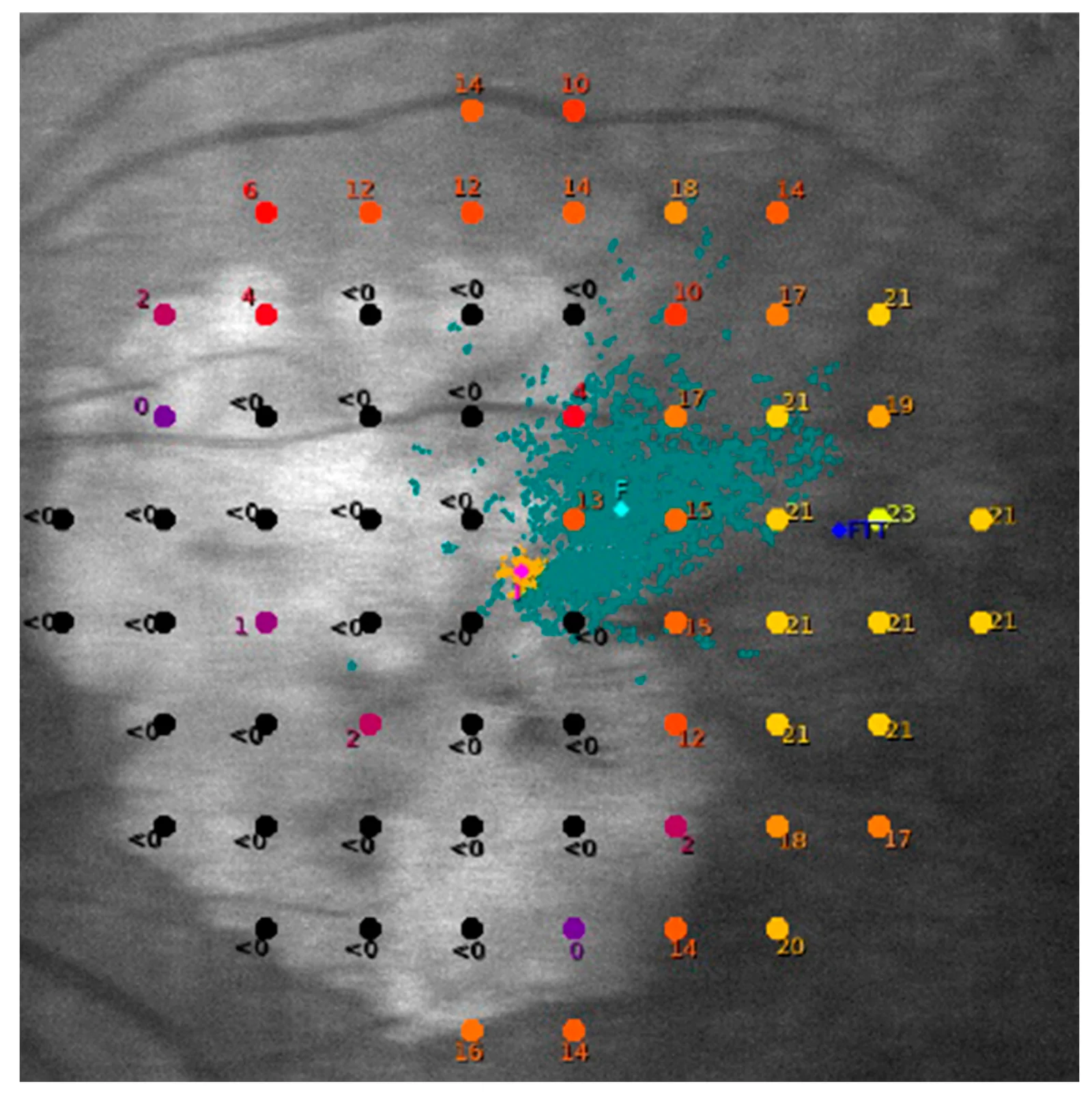
Microperimetry 10-2. The microperimetry image demonstrates a central scotoma with a preferred retinal locus (PRL) located at the temporal border of the defect. PRL is located at a retinal span of lower retinal sensitivity and unstable fixation. The functional training target (FTT—red circle) indicates the selected trained retinal locus (TRL), positioned within a region of higher retinal sensitivity intended to optimize functional vision.

The primary outcome was the performance during the training sessions (P1/P2) from sessions 1 to 5 (Figure 1). BT session performance was analyzed separately for P1 and P2, each using linear mixed-effects models, with session as a categorical fixed effect and a participant- specific random intercept. For P1 and P2, planned contrasts compared each subsequent session to session 1. Secondary outcomes were the within-person change in best-corrected distance visual acuity (BCVA, logMAR) of the trained eye from pre-BT baseline to the short- and long-term follow-up, binocular near visual acuity (logMAR), fixation stability calculated by the MAIA software version 1, 2.1 as the 63% bivariate contour ellipse area (BCEA, deg^2^), mean retinal sensitivity (Figure 2), and reading speed calculated by the MNRead app tested at 40 cm in words per minute.

For patients whose trained-eye BCVA worsened at long-term follow-up, paired baseline and long-term microperimetry examinations were reviewed, when available, for evidence of progression of visual-field loss. In eyes with central scotomas, the area of the seeing region within the analyzed microperimetry image was quantified in pixels^2^ using the same image-based measurement approach at baseline and follow-up. Change was calculated as follow-up minus baseline (post−pre), such that negative values indicated contraction of the seeing area. Additional microperimetric indicators of progression included change in the number of seeing points and mean retinal sensitivity. These measures were used to provide functional evidence of progression independent of BCVA. Because the image-based area measurement was not calibrated to retinal dimensions, values are reported in pixels^2^ and should be interpreted as within-eye relative changes rather than absolute retinal area measurements. Worsening BCVA was defined as an increase of ≥0.10 logMAR at long-term follow-up relative to baseline. This threshold was selected as the lower bound of the reported minimal clinically important difference for visual acuity (approximately 0.10–0.20 logMAR). We acknowledge that a 0.10-logMAR change may fall within the reported test–retest variability of visual acuity measurement; therefore, BCVA worsening was not considered, by itself, evidence of progression of the underlying disease. Available microperimetric and imaging findings were reviewed to provide additional evidence of disease progression independent of BCVA.

### 2.5. Statistical Analysis

Continuous variables are reported as mean (SD) or median (IQR), as appropriate, and categorical variables as counts and percentages. Longitudinal outcomes were analyzed using linear mixed-effects models in R (version 4.5.3).

Repeated measurements within participants were modeled using participant-specific random intercepts to account for within-participant correlation, with no additional residual covariance structure applied. Because this was a retrospective study reflecting routine clinical practice, not all outcome measures were performed at every visit, and some patients were lost to follow-up. Consequently, the number of available observations varied across participants, outcomes, and follow-up timepoints. Models accommodated incomplete longitudinal data under a missing-at-random assumption. Covariates included as fixed effects were age, sex, clinic, and type of vision loss (central, peripheral, diffuse, or nystagmus).

Model parameters were estimated using restricted maximum likelihood (REML), with Satterthwaite approximation used to estimate degrees of freedom. Models were assessed diagnostically using residual-versus-fitted plots, normal Q–Q plots, and checks for model convergence and singularity.

The primary performance measures (P1 and P2) obtained during BT were modeled longitudinally across BT sessions, with BT session as a categorial fixed effect.

Secondary visual outcome measures (BCVA, near vision, reading speed, BCEA and retinal sensitivity) were modeled longitudinally from baseline through follow-up, with visit type (baseline, short-term, long-term) and number of BT sessions received as fixed effects.

A two-sided α = 0.01 was prespecified before examination of the study results as a conservative criterion of significance. Unadjusted *p*-values and 95% confidence intervals are reported throughout.

## 3. Results

### 3.1. Cohort Characteristics

A total of 346 unique patients were included in the study with a mean age of 56.62 years ± 27.24 years, and sex distribution was balanced (51.7% vs. 48.3%; Table 1). Patients were drawn from two clinics in similar proportions (57.2% vs. 42.8%).

### 3.2. Primary Outcome—Within-Training Session Performance

P1 and P2 measures increased in parallel across sessions, reflecting progressively better TRL-centered fixation. Median P1 rose from 21.5% in session 1 to 32% in session 5, and P2 improved from 66% in session 1 to 81.5% in Session 5 (Table 2). Linear mixed-effects models demonstrated a significant effect of training on both measures. For P1, the effect of session was significant (F(4, 1073.6) = 21.64, *p* < 0.0001), with stepwise increases from Session 1 to Session 5 (Session 2: +3.28, 95% CI: 1.8–4.8; Session 3: +5.00, 95% CI: 3.5–6.5; Session 4: +6.53, 95% CI: 5.0–8.1; Session 5: +7.58, 95% CI: 4.9–10.3, all relative to Session 1; all *p* < 0.0001). For P2, the session effect was similarly significant (F(4, 1072.3) = 20.70, *p* < 0.0001), with progressive improvements (Session 2: +3.21, 95% CI: 1.6–4.8; Session 3: +5.65, 95% CI: 4.1–7.2; Session 4: +6.60, 95% CI: 5.0–8.2; Session 5: +7.18, 95% CI: 4.4–10.0 vs. Session 1; all *p* < 0.0001). Higher P1 and P2 values indicate that a greater proportion of fundus-tracked fixation points fell within the device-defined TRL-centered target windows during active training. The smaller number of observations in Sessions 4 and 5 primarily reflects variation in the number of training sessions completed in routine clinical practice, rather than increased task difficulty or reduced patient performance.

### 3.3. Secondary Outcomes

Distance visual acuity (Table 3) improved in the short term. Median VA at baseline was 0.40 logMAR (IQR 0.20–0.80, n = 343), at short-term follow-up 0.30 logMAR (IQR 0.10–0.70, n = 290), and 0.30 logMAR (IQR 0.10–0.80, n = 185) at long-term follow-up. Mixed-model analysis showed a significant improvement only at the short-term visit (F(2, 481.4) = 22.48, 95% CI: −0.083 to −0.045, *p* < 0.0001). Although the long-term median matched the short-term value, it was not significantly different from baseline (95% CI: −0.039 to 0.007, *p* = 0.177)—likely because greater variability at the long-term visit reduced statistical power despite similar medians.

Near visual acuity (Table 3) also improved over time. Median near VA at baseline was 0.20 logMAR (IQR 0.00–0.30, n = 311), at short-term follow-up 0.10 logMAR (IQR 0.00–0.30, n = 268), and 0.10 logMAR (IQR 0.00–0.30, n = 171) at long-term follow-up. Near vision showed a significant improvement at the short-term visit (F(2, 444.4) = 13.67, 95% CI: −0.059 to −0.027, *p* < 0.0001). In contrast, the long-term visit did not differ significantly from baseline (95% CI: −0.036 to 0.003, *p* = 0.094).

Baseline BCVA was the only significant predictor of final visual acuity following BT, with better initial vision associated with better visual outcomes. This association remained significant after adjustment for age and sex. In contrast, the type of vision loss (central, peripheral, diffuse, or nystagmus-associated) was not a significant predictor of outcome.

Median reading speed at baseline was 94.2 words per minute (IQR 65–139, n = 77), at short-term follow-up 93 words per minute (IQR 59.2–134, n = 63), and at long-term follow-up 114 words per minute (IQR 37–145, n = 21). Although median reading speed varied at both short-term and long-term follow-up, mixed-model analysis showed no significant effect of visit (F(2, 79.1) = 0.38, 95% CI: −3.963 to 9.950, *p* = 0.394 for short-term follow-up; 95% CI: −10.642 to 12.076, *p* = 0.900 for long-term follow-up), with neither follow-up timepoint differing significantly from baseline.

BCEA showed modest change over time. Median BCEA at baseline was 4.05°^2^ (IQR 0.9–11.33, n = 326), at short-term follow-up 2.8°^2^ (IQR 0.5–7.2, n = 229), and at long-term follow-up 3.4°^2^ (IQR 0.4–8.25, n = 115). However, mixed-model analysis did not identify a significant overall effect of visit on BCEA (F(2, 352.1) = 1.58, 95% CI: −1.981 to 0.410, *p* = 0.197 for short-term follow-up; 95% CI: −2.862 to 0.326, *p* = 0.119 for long-term follow-up). Analysis of PRL relocation after training was not possible due to insufficient data.

Retinal sensitivity remained largely stable over time. Median retinal sensitivity at baseline was 13.7 dB (IQR 7.4–18.6, n = 257), at short-term follow-up 13.6 dB (IQR 6.75–19.45, n = 175), and at long-term follow-up 14.35 dB (IQR 8.0–20.77, n = 90). Mixed-model analysis did not demonstrate a significant overall effect of visit on retinal sensitivity (F(2, 322.9) = 0.31, 95% CI: −0.829 to 1.728, *p* = 0.49 for short-term follow-up; 95% CI: −1.803 to 1.576, *p* = 0.895 for long-term follow-up).

### 3.4. Disease Progression and Exploratory Secondary Analysis

Fifty-two eyes were flagged clinically as worsened in the long term (AMD n = 14; glaucoma n = 9; retinal dystrophy n = 9; optic neuropathy n = 4; nystagmus n = 3; homonymous hemianopia n = 2; other n = 11). In paired analyses, central scotoma cases showed contraction of the seeing area, with the median seeing area decreasing from 2,276,435 pixels^2^ (IQR 789,611–6,404,369) at baseline to 1,857,856 pixels^2^ (IQR 656,336–3,384,732) at long-term follow-up (median change, −172,908 pixels^2^; n = 26; *p* = 0.045). This was accompanied by fewer seeing points (median change, −3; n = 26; *p* = 0.0011) and lower retinal sensitivity (median change, −1.50 dB; n = 24; *p* = 0.0008). BCEA was unchanged (median change, −0.48 deg^2^; n = 19; *p* = 0.418).

Tunnel-vision cases showed more variable area change (median −14,389 pixels^2^; n = 11; *p* = 0.139) with no median change in seeing points (0; n = 11; *p* > 0.99) and a small, non-significant RS reduction (−0.25 dB; n = 10; *p* = 0.314); BCEA remained stable (−0.08 deg^2^; n = 8; *p* = 0.461).

Across all worsened cases with paired data, seeing points (−1.5; n = 44; *p* = 0.0016) and retinal sensitivity (−1.20 dB; n = 40; *p* = 0.0005) declined, whereas BCEA did not show a consistent shift (median Δ −0.48 deg^2^; n = 32; *p* = 0.160).

An exploratory secondary analysis was conducted after excluding eyes with worsening BCVA during long-term follow-up. Because this subgroup was defined according to the subsequent visual acuity outcome, these findings are descriptive and should be interpreted cautiously because of potential selection bias. Median VA at baseline was 0.40 logMAR (IQR 0.20–0.80, n = 292), at short-term follow-up 0.30 logMAR (IQR 0.00–0.70, n = 241), and at long-term follow-up 0.20 logMAR (IQR 0.00–0.67, n = 134). Linear mixed-model analysis confirmed a significant effect of training on VA (F(2, 376.5) = 67.32), indicating meaningful improvement from baseline to both short- (95% CI: −0.083 to −0.054, *p* < 0.0001) and long-term (95% CI: −0.113 to −0.076, *p* < 0.001) follow-ups.

Near vision showed statistically significant improvements after training. Median near VA at baseline was 0.10 logMAR (IQR 0.00–0.30, n = 268), at short-term follow-up 0.00 logMAR (IQR 0.00–0.30, n = 224), and at long-term follow-up 0.00 logMAR (IQR 0.00–0.30, n = 130). Despite these similar distributions, the mixed-effects model detected a significant effect in short- (F(2, 354.5) = 14.90, 95% CI: −0.055 to −0.025, *p* < 0.0001) and long-term (95% CI: −0.048 to −0.011, *p* = 0.002) visits.

For the same cohort, reading speed remained stable across visits. Median reading speed at baseline was 100.95 wpm (IQR 66.0–139.25, n = 68), at short-term follow-up 97 wpm (IQR 62.0–138.59, n = 54), and at long-term follow-up 100.0 wpm (IQR 55.0–149.75, n = 16). The linear mixed model showed no significant change over time (F(2, 64.3) = 1.42, 95% CI: −1.432 to 11.236, *p* = 0.127 for short-term follow-up; 95% CI: −12.433 to 9.853, *p* = 0.818 for long-term follow-up).

BCEA values showed a trend toward improvement from baseline through short- and long-term follow-up. However, the change did not show a significant effect of visit on BCEA (F(2, 284.5) = 1.42, 95% CI −2.015 to 0.451, *p* = 0.213 for short-term follow-up; 95% CI −3.051 to 0.460, *p* = 0.148 for long-term follow-up). Median BCEA at baseline was 3.7°^2^ (IQR 0.80–10.55, n = 279), at short-term follow-up 2.5°^2^ (IQR 0.50–6.90, n = 197), and at long-term follow-up 2.4°^2^ (IQR 0.40–7.20, n = 85).

Retinal sensitivity remained stable across visits. Median sensitivity at baseline was 14.0 dB (IQR 7.8–18.90, n = 219), at short-term follow-up 13.55 dB (IQR 7.38–19.58, n = 150), and at long-term follow-up 15.0 dB (IQR 10.43–21.30, n = 64). Even though there was a trend toward higher median sensitivity at long-term, linear mixed-model analysis showed no significant effect of visit on retinal sensitivity (F(2, 274.9) = 0.39, 95% CI −0.926 to 2.067, *p* = 0.453 for short-term follow-up; 95% CI −1.413 to 2.846, *p* = 0.508 for long-term follow-up).

### 3.5. Safety Observations

Because this was a retrospective study, adverse events were not collected prospectively using a standardized safety questionnaire. Safety information was therefore based on symptoms documented in the clinical records during or following BT sessions. Transient dry-eye symptoms and mild headache were occasionally documented and resolved within hours or by the following day. No serious adverse events related to BT were documented. Accordingly, the absence of systematically collected safety data limits conclusions regarding the frequency of minor adverse events.

## 4. Discussion

In this large retrospective cohort of 346 patients undergoing MAIA-guided biofeedback training, we observed progressive improvements in fixation performance during training together with significant short-term improvements in both distance and near visual acuity. Importantly, eyes without evidence of disease progression maintained these gains over long-term follow-up, raising the hypothesis that deterioration in the full cohort could possibly reflect progression of the underlying disease rather than loss of the rehabilitation effect.

Progressive improvement in P1 and P2 across training sessions provides objective evidence of acquisition of the trained fixation strategy. These parameters quantify the proportion of fixation maintained within the target retinal locus during the rehabilitation and therefore serve as objective markers of oculomotor learning. Their consistent improvement over time suggests that patients progressively learned to stabilize fixation more effectively at the intended retinal location.

Although the median visual acuity improvement observed in our cohort was of approximately one line, this magnitude of change should not be dismissed as clinically trivial. In low-vision populations, even relatively small improvements in acuity can produce meaningful gains in functional independence, reading efficiency, face recognition, medication management, and overall quality of life. Previous studies have demonstrated significant associations between visual acuity and patient-reported quality of life, particularly among individuals with retinal disease [9,10,11]. Moreover, visual acuity thresholds around 20/50 to 20/70 frequently determine eligibility for driving privileges, occupational activities, and rehabilitation services in many jurisdictions. Consequently, a one-line improvement may represent the difference between maintaining or losing important aspects of independence. The functional importance of modest visual acuity gains is therefore likely greater in low-vision populations than in normally sighted individuals.

One notable finding was the apparent discrepancy between improvements in visual acuity and the absence of statistically significant changes in BCEA and retinal sensitivity. Several hypotheses may explain this observation. Visual acuity is a task-specific outcome that may improve through more efficient use of residual retinal function rather than fixation stability alone [12,13,14]. Furthermore, patients may learn to position visual targets more consistently within a small region of relatively preserved retina, thereby improving visual performance while producing only modest changes in BCEA. Second, BCEA is known to exhibit substantial interindividual variability, particularly in heterogeneous low-vision populations with different fixation strategies and underlying disease mechanisms. Finally, improvements in visual performance may reflect adaptive changes in oculomotor control and visual processing that are not fully captured by BCEA [8,15,16]. Although these mechanisms were not directly evaluated in the present study, they may contribute to improved visual function despite relatively stable global fixation metrics.

The subgroup analysis of eyes that experienced visual decline over time provides important clinical insight into the interpretation of long-term outcomes following BT. In these patients, worsening visual acuity was accompanied by objective evidence of disease progression, including enlargement of absolute scotomas or geographic atrophy and reduction in retinal sensitivity. These findings suggest that deterioration in visual performance may be more closely related to progression of the underlying disease process than to loss of training-induced adaptations. This distinction is clinically relevant because it supports the concept that BT remains beneficial even in progressive diseases, although the magnitude of improvement may eventually be offset by ongoing retinal degeneration. In such situations, repeat training sessions may be warranted to establish a new TRL and optimize the use of remaining retinal function as disease progression alters retinal architecture and microperimetry.

The biological plausibility of these findings is supported by an expanding body of evidence demonstrating substantial plasticity within both the oculomotor and visual systems following vision loss. Patients with central vision impairment frequently develop eccentric preferred retinal loci (PRLs) that function as substitutes for the damaged fovea. However, naturally selected PRLs are often not located in retinal regions that maximize visual performance [3,12,13,14,15]. Biofeedback training aims to guide patients toward a retinal locus with greater functional potential, such as higher retinal sensitivity and/or promoting more stable fixation, thereby improving the efficiency with which residual visual information is utilized. Functional neuroimaging studies further support this mechanism. Investigations in macular degeneration have demonstrated activation of cortical regions associated with the PRL stimulation, supporting the concept of adaptive cortical reorganization following central vision loss [17,18]. Furthermore, a randomized controlled trial in Stargardt disease demonstrated increased visual cortical activation following biofeedback training, providing evidence that rehabilitation-induced changes may extend beyond the retina to involve adaptive neural processes within the visual cortex [7]. Together, these findings support the hypothesis of biofeedback training as a rehabilitation strategy that may promote adaptive neuroplastic changes, thereby improving visual performance despite persistent structural retinal damage.

The recent EFFECT randomized trial by Crossland and colleagues reported no measurable benefit of biofeedback training [19]. However, important methodological differences may explain the discrepancy with our findings. The EFFECT protocol consisted of only two 10 min sessions (approximately 20 min in total), whereas patients in our program received a mean of 4.1 sessions lasting 20 min each (approximately 80–0 min in total). Similar training durations have been used in previous studies reporting favorable outcomes [5,6,8,20,21]. Differences in training dose and reinforcement may therefore contribute to the divergent results. Future prospective trials should directly evaluate the dose–response relationship and determine the optimal frequency and duration of biofeedback training.

Our findings have several practical implications for low vision rehabilitation. First, patients can reasonably be counseled to expect improvements in visual acuity following biofeedback training, even when conventional fixation metrics remain relatively unchanged. The magnitude of improvement appeared consistent across a broad range of ocular and neuro-ophthalmic conditions, suggesting that the underlying mechanism of rehabilitation may be applicable across different causes of vision loss.

Second, the attenuation of visual acuity gains observed in some patients at long-term follow-up raises the possibility that appeared to be associated primarily with progression of the underlying disease rather than loss of the acquired fixation strategy, may contribute to the observed decline. Accordingly, patients with progressive conditions may benefit from periodic reassessment and, where appropriate, additional training sessions. Whether scheduled booster sessions improve the durability of treatment effects remains unknown and should be evaluated prospectively.

Finally, biofeedback training should be viewed as one component of comprehensive vision rehabilitation. For patients whose primary goal is reading, combining fixation training with reading-specific interventions may provide greater functional benefit than either approach alone. Likewise, serial microperimetry and structural imaging may help distinguish disease progression from changes related to rehabilitation, allowing treatment strategies to be individualized over time.

## 5. Strengths and Limitations

This study has several strengths. It represents one of the largest cohorts of patients undergoing microperimetry-guided biofeedback training and includes a broad spectrum of low-vision disorders encountered in routine clinical practice. Multiple functional outcomes were evaluated using standardized assessments, and longitudinal analyses incorporated repeated measurements over both short- and long-term follow-up. In addition, structural imaging was reviewed in eyes demonstrating deterioration, allowing progression of the underlying disease to be distinguished from potential loss of treatment effect.

Several limitations should also be acknowledged. The retrospective design and absence of a control group preclude causal inference. Attrition at longer follow-up intervals, particularly for reading speed, may have introduced bias despite the use of linear mixed-effects models to accommodate incomplete longitudinal data. Because this study reflected routine clinical practice, not all outcome measures were performed at every visit, resulting in variability in the availability of measurements across participants and follow-up timepoints. The secondary long-term analysis excluding eyes with documented disease progression may also have introduced selection bias. This analysis should be interpreted as hypothesis-generating rather than as definitive evidence that disease progression accounted for the attenuation of long-term visual acuity improvement. Also, the heterogeneous study population and variable number of training sessions likely increased variability in treatment response. Furthermore, we relied on summary fixation metrics exported by the microperimeter rather than continuous eye movement recordings, limiting assessment of more subtle oculomotor adaptations. Contrast sensitivity, vision-related quality-of-life measures, and detailed analyses of PRL relocation were not consistently available and therefore could not be evaluated. These outcomes should be incorporated into future prospective studies.

Although the heterogeneous cohort enhances the generalizability of our findings, it may also have obscured diagnosis-specific treatment effects. It is likely that different disease mechanisms respond differently to biofeedback training. Future adequately powered prospective studies should investigate disease-specific outcomes and identify clinical predictors of successful rehabilitation.

Future randomized controlled trials should compare biofeedback training with sham or standard rehabilitation, evaluate the optimal training dose and reinforcement schedule, and incorporate reading-specific interventions where appropriate. Longitudinal assessment of PRL or trained retinal locus relocation, retinal sensitivity, structural progression, contrast sensitivity, and patient-reported vision-related quality of life will provide a more comprehensive understanding of treatment effects. In addition, emerging evidence that cortical activation is not restricted to a single PRL suggests that rehabilitation strategies targeting multiple retinal loci with preserved function may warrant investigation as an alternative to traditional single-locus approaches [7].

## 6. Conclusions

Microperimetry-guided biofeedback training was associated with significant short-term improvements in best-corrected visual acuity and objective indicators of fixation learning across a heterogeneous cohort of patients with low vision. Although the observed long-term visual acuity decline was accompanied by evidence of underlying disease progression in some patients, it is not possible to determine the extent to which disease progression, loss of the trained fixation strategy, or both contributed to this decline. These findings support the role of biofeedback training as a valuable component of multidisciplinary vision rehabilitation and reinforce the biological plausibility of neuroplastic adaptation following vision loss. Prospective randomized studies are now needed to define the optimal treatment dose, identify patients most likely to benefit, and determine strategies to maximize the durability of functional improvements.

## Figures and Tables

**Table 1:** Participant characteristics at baseline (2017–2023; n = 346).

Variable	Mean	SD
Age, years	56.62	27.24
Short-term follow-up interval, months	1.3	1.5
Long-term follow-up interval, months	18.7	11.3
BT sessions per patient	4.1	0.6
Sex	**n**	**%**
Female	179	51.7
Male	167	48.3
Diagnosis at baseline		
AMD	65	
Hemianopia/quadrantanopia	65	
Optic neuropathy	50	
Nystagmus	45	
Glaucoma	35	
Retinal dystrophy	30	
Other visual-field defects	14	
Other retinopathies	7	
Uveitis	6	
Myopic degeneration	6	
Macular hole	5	
Foveal hypoplasia	4	
Diabetic retinopathy/DME	4	
ERM	3	
Oculocutaneous albinism (OCA)	3	
Congenital cataract	2	
Corneal disease	1	
Amblyopia	1	

BT = biofeedback training; AMD = Age-Related Macular Degeneration; DME = Diabetic Macular Edema; ERM = Epiretinal Membrane.

**Table 2:** Session performance during biofeedback training.

Session	n (Paired)	Median P1 (IQR) (%) *	Median P2 (IQR) (%) *
1	346	21.5 (9–42)	66.0 (35–91)
2	346	25.0 (10–45)	69.5 (39.25–93)
3	339	27.0 (13–48.5)	72.0 (45.5–95)
4	312	30.0 (13–52)	74.0 (47.75–94.25)
5	76	32.0 (18–57.5)	81.5 (55.75–95.25)

* P1 and P2 are MAIA device-derived in-session fixation-control metrics recorded during active biofeedback training. P1 is the percentage of fundus-tracked fixation points within the 1° radius (2° diameter) of the trained retinal locus (TRL). P2 is the percentage within the broader target window (2° radius/4° diameter). Higher values indicate a greater proportion of training time spent fixating within the intended TRL-centered window. Sessions 1–5 refer to sequential BT sessions, not separate clinical visual function tests. Lower n in Sessions 4–5 reflects incomplete completion of later sessions in routine clinical workflow.

**Table 3:** Distance and near best corrected visual acuity across visits.

Outcome	Baseline (logMAR + IQR)	Short Term	n	95% CI	*p*-Value	Long Term	n	95% CI	*p*-Value
BCVA	0.40 (0.20–0.80)	0.30 (0.10–0.70)	290	−0.083 to −0.045	<0.0001	0.30 (0.10–0.80)	185	−0.039 to 0.007	0.177
Near vision	0.20 (0.00–0.30)	0.10 (0.00–0.30)	268	−0.059 to −0.027	<0.0001	0.10 (0.00–0.30)	171	−0.036 to 0.003	0.094
BCVA (secondary analysis)	0.40 (0.20–0.80)	0.30 (0.00–0.70)	241	−0.083 to −0.054	<0.0001	0.20 (0.00–0.67)	134	−0.113 to −0.076	<0.0001
Near vision (secondary analysis)	0.10 (0.00–0.30)	0.00 (0.00–0.30)	224	−0.055 to −0.025	<0.0001	0.00 (0.00–0.30)	130	−0.048 to −0.011	0.002

## Data Availability

The data presented in this study are not publicly available due to ethical and privacy restrictions related to the protection of patient confidentiality. The data may be available from the corresponding author upon reasonable request and subject to approval by the appropriate institutional and research ethics authorities.
